# Nasal polyps, aspirin sensitivity, and late onset asthma are crucial to identify severe asthma. clinicaltrials.gov id : nct01513837

**DOI:** 10.1186/2045-7022-5-S2-O3

**Published:** 2015-03-23

**Authors:** José A Castillo, Vicente Plaza, Gustavo Rodrigo, Berta Juliá, César Picado, Joaquim Mullol

**Affiliations:** 1 Hospital Universitario Quirón Dexeus, Barcelona, Spain; 2 Hospital Santa Creu i Sant Pau, Respiratory Medicine, Barcelona, Spain; 3 Hospital de las Fuerzas Armadas, Respiratory Medicine, Montevideo, Uruguay; 4 MSD-España, Departamento Médico, Madrid, Spain; 5 Hospital Clinic, Respiratory Medicine, Barcelona, Spain; 6 Hospital Clinic, Immunoallèrgia Respiratòria Clínica i Experimenta, Barcelona, Spain

## Background

Early recognition of severe asthma may improve its management, modify its natural history by better approaching therapy and helps identifying patients resistant to current asthma therapy. Rhinitis and chronic rhinosinusitis are linked to asthma by clinical, epidemiological and similar inflammatory mechanisms which interrelates upper and lower airways. Evidence for high rate of chronic rhinosinusitis has been related to severe asthma.

## Objective

We sought to investigate the differential clinical features which may identify severe asthma under the United Airways concept.

## Methods

A prospective study carried out in 2010-2011 by pulmonologists and ENT specialists in 23 centers from Spain and Latinamerica, asthmatics patients (N=492); age 45(±15) yo; 70.5% female were recruited according to GINA: 17.3% intermittent and 82.7% persistent (24.6% mild, 31.4% moderate, and 26.7% severe). The presence of allergic (AR) and non-allergic (NAR) rhinitis and chronic rhinosinusitis with (CRSwNP) and without nasal polyps (CRSsNP) evaluated according to ARIA and EPOS, by nasal symptoms, prick test, nasal endoscopy and sinus CT scan. Asthma control was assessed by Asthma Control Test (ACT) while aspirin intolerance (AIA) by clinical history and/or aspirin challenge.

## Results

More than one third (35%) of severe asthma patients, predominantly non-atopic (44%), reported CRSwNP (OR:3.4; p<0.001), and 30% AR (OR:1.03; NS). Aspirin sensitivity was associated with severe asthma (43%; OR:7.8; p<0.05) The presence of CRSwNP was also associated with AIA (38.9%; OR:9.05; p<0.001). Apart from high asthma symptom score, need of intense treatment, and exacerbation rate, patients with severe asthma were older (p<0.001), had late onset asthma (p<0.001), and showed higher sinus CT scores (p<0.001) which correlated with poor asthma control. Multiple logistic regression analysis showed that CRSwNP, AIA, and late onset asthma were independently associated with severe asthma.

## Conclusion

Chronic rhinosinusitis with nasal polyps, aspirin sensitivity, and late onset asthma are crucial clinical features and associations that help to identifying severe asthma.

